# Progressive familial intrahepatic cholestasis

**DOI:** 10.1186/1750-1172-4-1

**Published:** 2009-01-08

**Authors:** Anne Davit-Spraul, Emmanuel Gonzales, Christiane Baussan, Emmanuel Jacquemin

**Affiliations:** 1 Biochemistry, Bicêtre Hospital, University of Paris-sud XI, Assistance Publique-Hôpitaux de Paris, Paris, France; 2 Pediatric Hepatology and National Reference Centre for Biliary Atresia, Bicêtre Hospital, University of Paris-sud XI, Assistance Publique-Hôpitaux de Paris, Paris, France; 3 INSERM, UMR-S757, University of Paris-sud XI, Orsay, France

## Abstract

Progressive familial intrahepatic cholestasis (PFIC) refers to heterogeneous group of autosomal recessive disorders of childhood that disrupt bile formation and present with cholestasis of hepatocellular origin. The exact prevalence remains unknown, but the estimated incidence varies between 1/50,000 and 1/100,000 births.

Three types of PFIC have been identified and related to mutations in hepatocellular transport system genes involved in bile formation. PFIC1 and PFIC2 usually appear in the first months of life, whereas onset of PFIC3 may also occur later in infancy, in childhood or even during young adulthood. Main clinical manifestations include cholestasis, pruritus and jaundice. PFIC patients usually develop fibrosis and end-stage liver disease before adulthood. Serum gamma-glutamyltransferase (GGT) activity is normal in PFIC1 and PFIC2 patients, but is elevated in PFIC3 patients. Both PFIC1 and PFIC2 are caused by impaired bile salt secretion due respectively to defects in *ATP8B1 *encoding the FIC1 protein, and in *ABCB11 *encoding the bile salt export pump protein (BSEP). Defects in *ABCB4*, encoding the multi-drug resistant 3 protein (MDR3), impair biliary phospholipid secretion resulting in PFIC3.

Diagnosis is based on clinical manifestations, liver ultrasonography, cholangiography and liver histology, as well as on specific tests for excluding other causes of childhood cholestasis. MDR3 and BSEP liver immunostaining, and analysis of biliary lipid composition should help to select PFIC candidates in whom genotyping could be proposed to confirm the diagnosis. Antenatal diagnosis can be proposed for affected families in which a mutation has been identified. Ursodeoxycholic acid (UDCA) therapy should be initiated in all patients to prevent liver damage. In some PFIC1 or PFIC2 patients, biliary diversion can also relieve pruritus and slow disease progression. However, most PFIC patients are ultimately candidates for liver transplantation. Monitoring of hepatocellular carcinoma, especially in PFIC2 patients, should be offered from the first year of life. Hepatocyte transplantation, gene therapy or specific targeted pharmacotherapy may represent alternative treatments in the future.

## Disease name and synonyms

PFIC = Progressive familial intrahepatic cholestasis

PFIC1 = Progressive familial intrahepatic cholestasis type 1 = Byler disease = FIC1 deficiency

PFIC2 = Progressive familial intrahepatic cholestasis type 2 = Byler syndrome = BSEP deficiency

PFIC3 = Progressive familial intrahepatic cholestasis type 3 = MDR3 deficiency

## Definition

**Figure 1:**
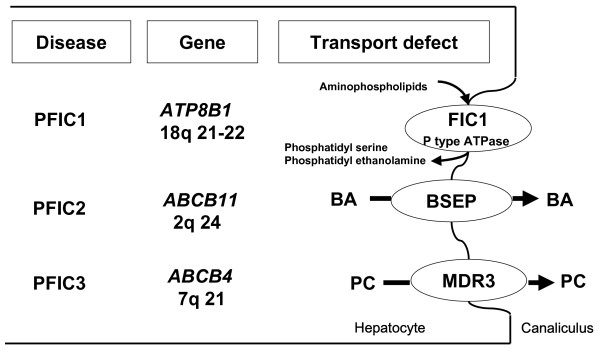
**Progressive Familial Intrahepatic Cholestasis (PFIC): Types, related genes, and transport defects**. BA: bile acid; PC: phosphatidylcholine.

Progressive familial intrahepatic cholestasis (PFIC) refers to heterogeneous group of autosomal recessive liver disorders of childhood in which cholestasis of hepatocellular origin often presents in the neonatal period or first year of life and leads to death from liver failure at ages usually ranging from infancy to adolescence [1,2]. Recent molecular and genetic studies have allowed the identification of genes responsible for three types of PFIC and have shown that they were related to mutations in hepatocellular transport system genes involved in bile formation (Figure 1).

## Epidemiology

In our experience, PFIC represents 10 to 15% of causes of cholestasis in children and 10 to 15% of liver transplantation indications in children. PFIC1 and PFIC2 represent 2/3 of cases of PFIC, and PFIC3 1/3 of cases [3]. The true incidence of PFIC is not precisely known, but PFIC is considered a rare disease with an estimated incidence of 1/50,000 to 1/100,000 births. All types of PFIC exist worldwide. Both sexes seem to be equally affected.

## Clinical description

Main characteristics of PFIC 1–3 are summarized in Table 1.

**Table 1:** Main characteristics of Progressive Familial Intrahepatic Cholestasis (PFIC)

	PFIC1 (Byler's disease)	PFIC2 (BSEP deficiency)	PFIC3 (MDR3 deficiency)
Transmission	autosomal recessive	autosomal recessive	autosomal recessive

Pruritus	severe	severe	moderate

Serum GGT activity	normal	normal	high

Ductular proliferation	absent	absent	present

Serum primary bile acid concentration	very high	very high	high

Bile composition	low primary bile acid concentration	very low primary bile acid concentration	low phospholipid concentration

Chromosomal locus	18q21-22	2q24	7q21

Gene/protein	*ATP8B1* FIC1	*ABCB11* BSEP	*ABCB4* MDR3

Hepatocyte location	canalicular membrane	canalicular membrane	canalicular membrane

Other sites of expression	Cholangiocytes Intestine, Pancreas	none	none

Functional defect	ATP-dependent aminophospholipid transport	ATP-dependent bile acid transport in bile	ATP-dependent phosphatidylcholine translocation in bile

Cholestasis is a major clinical sign in all PFIC forms.

It usually appears in the first months of life in patients with PFIC1, and is characterized by recurrent episodes of jaundice, which become permanent later in the course of the disease. In case of PFIC2, the initial presentation and the evolution seem to be more severe, with permanent jaundice from the first months of life and rapid appearance of liver failure within the first years of life. Early hepatocellular carcinoma (before one year of age) may complicate the course of PFIC2. Severe pruritus is usually observed in PFIC1 and PFIC2 patients. Phenotypic findings in PFIC1 and PFIC2 are similar, but some slight phenotypic difference have been identified [1,4-8]. Extrahepatic features that have been reported in PFIC1 patients (persistent short stature, sensorineural deafness, watery diarrhea, pancreatitis, elevated sweat electrolyte concentration, liver steatosis) [9], have not been described in PFIC2. In PFIC3, clinical signs of cholestasis are noted within the first year of life in about one third of patients and rarely in the neonatal period, in contrast to PFIC1 and 2. PFIC3 may also manifest later in infancy, in childhood or even in young adulthood. Gastrointestinal bleeding due to portal hypertension and cirrhosis is the presenting symptom in adolescent or young adult patients. Pruritus is usually mild. Evolution is characterized by chronic icteric or anicteric cholestasis, portal hypertension and liver failure. In half of the patient, liver transplantation is required at a mean age of 7.5 years. No liver tumor has yet been reported in association with PFIC3 [10].

## Laboratory findings

Patients with PFIC1 and PFIC2 have normal serum gamma-glutamyltransferase (GGT) activity, normal serum cholesterol level and very high serum bile acid concentration. Although PFIC1 and PFIC2 share similar laboratory findings, PFIC2 patients have higher transaminase and alpha-fetoprotein serum levels at diagnosis than those with PFIC1 [1,4-9]. Patients with PFIC3 have a persistent high serum GGT activity, normal serum cholesterol level and moderately raised concentrations of serum primary bile salts.

## Histological findings

**Figure 2:**
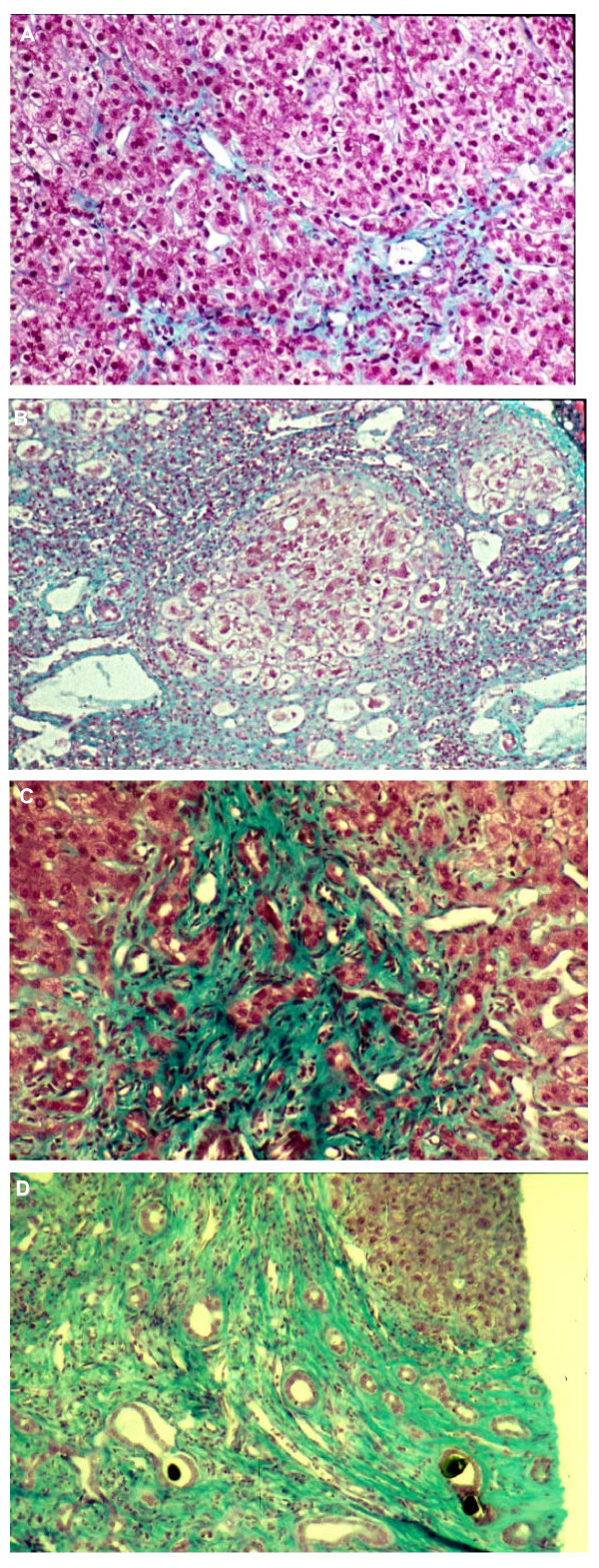
**Liver histology**. A: liver histology in a PFIC1 patient at age six months. It shows moderate lobular and portal fibrosis; B: liver histology in a PFIC2 patient at age 11 months. It shows hepatocellular necrosis, giant cell transformation and cirrhosis; C: liver histology in a PFIC3 patient at age 10 months. It shows strong (true) ductular proliferation within the all portal tract; D: at age 5 years in the same PFIC3 patient, there is a typical picture of biliary cirrhosis with ductules containing bile plugs, while patent biliary tree.

In PFIC1 patients, liver histology is characterized by canalicular cholestasis and the absence of true ductular proliferation with only periportal biliary metaplasia of hepatocytes. In PFIC2 patients, the same signs are seen but the liver architecture is more perturbed, with more pronounced lobular and portal fibrosis and inflammation. Hepatocellular necrosis and giant cell transformation is also much more pronounced in PFIC2 than in PFIC1 and may persist with time. These differences between PFIC1 and PFIC2 likely reflect the severe lobular injury present in PFIC2 (Figure 2A–B) [1,8,9].

In PFIC3, liver histology, obtained at the time of diagnosis (Figure 2C), shows portal fibrosis and true ductular proliferation with mixed inflammatory infiltrate. In a few instances, cholestasis is present in the lobule and in some ductules containing bile plugs. Slight giant transformation of hepatocytes can be observed. Cytokeratin immunostaining confirms the strong ductular proliferation within the portal tract. At a later stage (Figure 2D), there is extensive portal fibrosis and a typical picture of biliary cirrhosis. Interlobular bile ducts are seen in most portal tracts and there is neither periductal fibrosis nor biliary epithelium injury [11].

## Etiology

### PFIC 1

The first type, called PFIC1 (Byler disease) is caused by mutations in the ATP8B1 gene (also designated FIC1) [6,12]. This gene, which encodes a P-type ATPase, is located on human chromosome 18 and is also mutated in the milder phenotype, benign recurrent intrahepatic cholestasis type 1 (BRIC1) and in Greenland familial cholestasis [6,12,13]. FIC1 protein is located on the canalicular membrane of the hepatocyte but within the liver it is mainly expressed in cholangiocytes [14-16]. The function of this P-type ATPase is unknown but it could be an aminophospholipid transporter responsible for maintaining the enrichment of phosphatidylserine and phosphatidylethanolamine on the inner leaflet of the plasma membrane in comparison of the outer leaflet [6,14]. The asymmetric distribution of lipids in the membrane bilayer plays a protective role against high bile salt concentrations in the canalicular lumen [17]. How mutations in this protein cause cholestasis is unclear. It is postulated that abnormal protein function might indirectly disturb the biliary secretion of bile acids, explaining the low biliary bile acid concentration found in PFIC1 patients [4,6]. Different studies have shown that impaired ATP8B1 function in patients with PFIC1 results in substantial down regulation of FXR, a nuclear receptor involved in the regulation of bile acid metabolism with subsequent downregulation of BSEP in the liver and upregulation of bile acid synthesis, and of the apical sodium bile salt transporter (ASBT) in the intestine [16,18,19]. All in all, these events lead to hepatocyte bile acid overload. Moreover, evidence for cystic fibrosis transmembrane conductance regulator (CFTR) down regulation in cholangiocytes has also been reported in patients with PFIC1 which could contribute to impairment of bile secretion and explain some extrahepatic features [16].

The *ATP8B1 *gene is expressed in various organs, including the liver, pancreas, kidney and small intestine, but is more highly expressed in the small intestine than in the liver [12]. Therefore, it is thought to also be involved in the enterohepatic cycling of bile salts. This may also explain the chronic diarrhea present in a few children with PFIC1. Other extrahepatic features associated with PFIC1 such as persistent short stature, deafness and pancreatitis suggest a general cell biological function for FIC1 [6,9,20].

It is very likely that FIC1 disease represents a continuum with intermediate phenotypes between the benign phenotype BRIC1 and the severe phenotype PFIC1 [6]. So far, there is no clear explanation for the phenotypic difference between patients with BRIC1 and those with PFIC1. Mutation analyses suggest that mutations identified in patients with PFIC1 would severely disrupt protein function, whereas protein function would be only partially impaired in patients with BRIC1 [21]. Genotype-phenotype associations will probably be complicated because dramatic variability in phenotypic presentation has already been identified in patients with BRIC1 with a common mutation. In addition, many patients with FIC1 disease are compound heterozygous, which will also complicate the identification of genotype-phenotype correlations [21]. Heterozygous *ATP8B1 *mutations have also been identified in some cases of intrahepatic cholestasis of pregnancy (ICP), that can be referred to under the term ICP type 1 (ICP1) [22,23].

### PFIC 2

The second type, called PFIC2, is caused by mutations in the *ABCB11 *gene (also designated *BSEP*) [7,24]. Initially, PFIC2 was reported under the name Byler syndrome [1,4]. The *ABCB11 *gene encodes the ATP-dependent canalicular bile salt export pump (BSEP) of human liver and is located on human chromosome 2. BSEP protein, expressed at the hepatocyte canalicular membrane, is the major exporter of primary bile acids against extreme concentration gradients. Mutations in this protein are responsible for the decreased biliary bile salt secretion described in affected patients, leading to decreased bile flow and accumulation of bile salts inside the hepatocyte with ongoing severe hepatocellular damage.

BSEP deficiency represents also a phenotypic continuum between BRIC2 and PFIC2. So far, no clear genotype-phenotype correlation has been identified among PFIC2 patients, but most children with BSEP mutations, regardless of the mutation type, had no canalicular BSEP protein expression [5]. Severe phenotypes are often associated with mutations leading to premature protein truncation or failure of protein production. Insertion, deletion, non sense and splicing mutations result in damaging effects and patients exhibited little or no detectable BSEP at the hepatocyte canaliculus. Missense mutations are also common defects [25] that either affect protein processing and trafficking (*i.e. *p.E297G, p.D482G) [26,27] or disrupt functional domains and protein structure. Thus detectable BSEP expression (*i.e. *p.N490D, p.G562D, p.R832C, p.A1110E) does not exclude functional BSEP deficiency. Some mutations have been functionally characterized confirming the defect of bile acid secretion [28,29].

In milder disease, such as BRIC2 [30], missense mutations predominate over those leading to failure of protein production and mutations occur in less conserved regions than in the NBFs (nucleotide binding fold) which contain the Walker A/B motifs and ABC signature. Cholelithiasis was also reported in BRIC2 patients [[30,31] and E. Jacquemin personal data]. Heterozygous *ABCB11 *mutations have also been identified in cases of ICP (ICP2) [32], drug induced cholestasis [33] and transient neonatal cholestasis [34].

### PFIC 3

The third type of PFIC, called PFIC3, is caused by a genetic defect in the *ABCB4 *gene (also designated *MDR3*) located on chromosome 7. Class III Multidrug Resistance (MDR3) P-glycoprotein (P-gp), is a phospholipid translocator involved in biliary phospholipid (phosphatidylcholine) excretion and is predominantly, if not exclusively, expressed in the canalicular membrane of the hepatocyte [10]. Cholestasis results from the toxicity of bile in which detergent bile salts are not inactivated by phospholipids, leading to bile canaliculi and biliary epithelium injuries. The mechanism of liver damage in PFIC3 patients is likely related to the absence of biliary phospholipids [11]. Injury to bile canaliculi and biliary epithelium results probably from continuous exposure to hydrophobic bile salts, the detergent effects of which are no longer countered by phospholipids, leading to cholangitis [11]. In addition, the stability of mixed micelles in bile is determined by a three-phase system, in which a proper proportion of bile salts and phospholipids are necessary to maintain solubility of cholesterol. The absence of phospholipids in bile would be expected to destabilize micelles and promote lithogenicity of bile with crystallization of cholesterol, which could favor small bile duct obstruction. These cholangiopathy mechanisms fit well with the histologic findings such as ductular proliferation. PFIC3 represents an important example of canalicular transport defect that leads to the development of cholangiopathy.

The phenotypic spectrum of PFIC3 ranges from neonatal cholestasis to cirrhosis in young adults [11,35]. In our experience, *ABCB4 *sequence analysis in 50 PFIC3 patients revealed around 30 different *ABCB4 *mutations. Mutations were characterized on both alleles in most cases. In one third of cases, mutations gave rise to a truncated protein. When tested, no MDR3 P-glycoprotein could be detected by immunostaining in the liver of these patients. The absence of MDR3 protein can be explained in two ways. The truncated protein may be broken down very rapidly after synthesis giving rise to extremely low steady state levels of the protein. More likely, the premature stop codon may lead to instability and decay of the *ABCB4 *mRNA. This latter explanation is supported by the near absence of *ABCB4 *mRNA in northern blottings on liver samples from several patients [36,37]. The remaining 2/3 of patients had missense mutations. Some of them were found in the highly conserved aminoacids sequences of the Walker A and B motifs which are involved in ATP-binding [38]. Such aminoacid changes in the Walker A or B motif are generally not compatible with ATPase activity and transport processes [38-41]. Alternatively, missense mutations might result in intracellular misprocessing of MDR3 as shown for other ABC transporters [27,42-44]. Indeed, such missense mutations were associated with a decreased level of MDR3 canalicular protein [44]. Whatever the mechanism involved, the low level of biliary phospholipids found in patients with missense mutations demonstrates the MDR3 functional defect [11]. There is now strong evidence that in addition to PFIC3, an MDR3 defect can be involved in ICP (ICP3) [32,43,45-48], cholesterol gallstone disease [49-52] and drug induced cholestasis [53-55]. A MDR3 defect is also likely to be involved in some cases of transient neonatal cholestasis and adult idiopathic cirrhosis [10,35,56]. MDR3 deficiency may also represent a clinical continuum, as a single patient may experience different phenotypes during the disease course, starting with cholesterol cholelithiasis, then ICP and ending with biliary cirrhosis [57].

## Diagnosis and diagnostic methods

**Figure 3:**
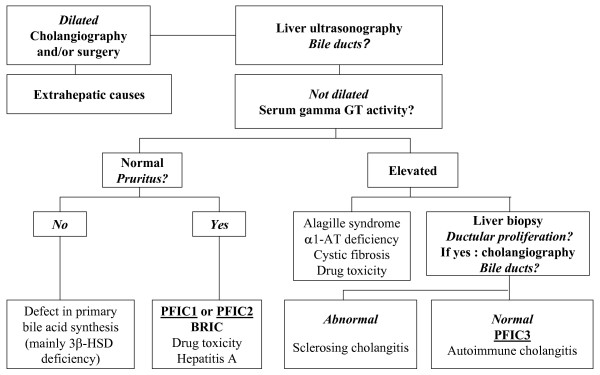
**Schematic approach to the diagnosis of PFIC excluding the neonatal period, during which biliary atresia is the main cause of cholestasis**.

PFIC should be suspected in children with a clinical history of cholestasis of unknown origin after exclusion of other main causes of cholestasis, (*e.g. *biliary atresia, Alagille syndrome, alpha1antitrypsine deficiency, cystic fibrosis, sclerosing cholangitis and extrahepatic bile duct obstruction) [11]. High serum bile acid concentration excludes primary disorders of bile acid synthesis [58]. Patients with PFIC1 and PFIC2 have normal serum GGT activity, while patients with PFIC3 have high serum GGT activity. PFIC3 patients can also be distinguished from patients with the other types of PFIC (PFIC1 and PFIC2) in that they present rarely with cholestatic jaundice at the neonatal period, but rather later in infancy, in childhood or in young adulthood. A schematic approach to the diagnosis of PFIC is proposed in Figure 3. This combined clinical, biochemical, radiological and histological approach associated with liver immunostaining and biliary lipid analysis, should help to select PFIC candidates in whom a molecular diagnosis can be proposed.

## Diagnostic methods

### • Radiological approach

Initial ultrasonography of the liver is performed to exclude biliary tract disease. Typically, ultrasonography is normal but may reveal a huge gallbladder. Sometimes, biliary stones may be identified. When performed, cholangiography shows a normal biliary tree, excluding sclerosing cholangitis, and allows bile to be collected for biliary lipid analysis [11].

### • Liver histology

is important for diagnosis. It also allows liver immunostaining to be performed. Typical features were described in the clinical description section and are shown in Figure 2. When signs of biliary obstruction are seen at liver biopsy, as may occur in PFIC3, cholangiography must be performed to exclude sclerosing cholangitis [11,59].

### • Liver immunostaining

Commercially available MDR3 and BSEP antibodies allow liver immunostaining to be performed [5,11,25]. Absence of canalicular or mild immunostaining are in favor of a gene defect. Normal staining does not exclude a gene defect as a mutation may induce a loss of function but normal synthesis and addressing.

### • Electron microscopy

Typical ultrastructural aspects of bile may allow differentiation of PFIC1 and PFIC2 but liver ultrastructural histology is not easily available [4,60].

### • Biliary lipid analysis

Biliary lipid analysis is performed on gallbladder bile or on bile collected by duodenal aspiration (pure choledochal bile). In case of gallbladder punction, bile contamination by blood may falsify bile analysis. In case of duodenal aspiration, bile dilution or bile contamination by alimentary phospholipids may falsify bile analysis. The biliary bile salt concentration is dramatically decreased (<1 mM) in PFIC2 patients [5] and only mildly decreased in PFIC1 patients (3–8 mM) [[9] and A. Davit-Spraul, personal data]. The normal concentration of biliary primary bile salts distinguishes PFIC3 patients from those with PFIC1 and PFIC2 [4,5,11,58]. In PFIC3 patients, the biliary phospholipid level is dramatically decreased (1–15% of total biliary lipids; normal range 19–24%). Biliary bile salt:phospholipid and cholesterol:phospholipid ratios are approximately 5-fold higher than in wild type bile. The residual percentage of biliary phospholipids seems directly related to the severity of *MDR3 *mutation and consequently to the residual activity of the MDR3 P-glycoprotein. Patients with "severe" mutations (nonsense, frameshift) have a low percentage of biliary phospholipids <2%, while patients with missense mutations have a high percentage of biliary phospholipids ≥ 2%. In our experience, the threshold that predicts a positive response to ursodeoxycholic acid (UDCA) therapy in PFIC3, is represented by a percentage of biliary phospholipids of around 7%. The combination of abnormal MDR3 canalicular immunostaining and low percentage of biliary phospholipids is highly suggestive of MDR3 deficiency.

### • Molecular analysis

PFIC1: *ATP8B1 *gene (coding exons 2–28)

PFIC2: *ABCB11 *gene (coding exons 2–28)

PFIC3: *ABCB4 *gene (coding exons 2–28)

Gene analysis is usually performed by DNA sequencing of the 27 coding exons and their splice junctions. In case of silent or intronic mutations, a *in silico *test is helpful to predict a potential splice defect which must be confirmed by RNA analysis (RT-PCR and sequencing) [61]. The use of a resequensing chip dedicated to genetic cholestasis could facilitate identification of gene mutation [62].

### • ^31^P MRS spectroscopy

It remains to be evaluated if ^31^P MRS (Magnetic Resonance Spectroscopy) would be a valuable non-invasive tool to characterize canalicular transport defects, such as was suggested for ICP1 [22].

## Differential diagnosis

Several other autosomal recessive PFIC-like diseases are known. Liver diseases resembling PFIC with normal GGT activity have been identified as inborn errors in primary bile acid synthesis and represent distinct disorders [58]. Other liver diseases resembling PFIC with normal serum GGT activity are recognized. Familial Amish hypercholanemia represents a PFIC-like disorder not due to a primary defect of the transport system involved in bile formation, but to a tight junction protein defect combined with a defect of primary bile acid conjugation [63,64]. Cholestasis is due to impaired transport of unconjugated bile acids into bile and to bile leakage into plasma through abnormal canalicular tight junctions increasing paracellular permeability. Another category of progressive cholestastic liver disease of childhood could be due to abnormal villin expression, leading to loss of structural integrity of canalicular microvilli impairing biliary secretion system function [65]. A case of hypercholanemia due to mutations in the m-epoxide hydrolase gene has also been reported (*EPHX1*) [66,67]. Arthrogryposis-renal dysfunction cholestasis syndrome is a complex disease due to mutation of *VPS33B *involved in intracellular trafficking and targeting of apical proteins. The gene defect results in a loss of apical protein expression in the liver and kidneys [68]. From a liver point of view, it represents another PFIC-like disorder with normal GGT activity. In cases of high serum GGT activity, sclerosing cholangitis must be excluded [59,69]. North American Indian Childhood Cirrhosis due to a defect in the cirrhin gene is characterized by high GGT activity [70] as is Aagenes syndrome, a lymphedema cholestasis syndrome of unknown cause [71].

## Genetic counseling and antenatal diagnosis

Genotyping should be used to confirm the diagnosis of PFIC in affected children. Heterozygosity of parents for the defects found in affected patients confirmed the recessive inheritance of the disease. This understanding has already allowed prenatal diagnosis. Antenatal diagnosis of PFIC requires clinical and biochemical expertise [56].

## Specific management including treatment

Therapy with UDCA should be considered in the initial therapeutic management of children with all types of PFIC [72]. UDCA therapy may be effective in some patients, especially those with PFIC3 with missense mutations who have less severe disease in comparison to children with a mutation leading to a truncated protein [11].

Some patients with PFIC1 or PFIC2 may also benefit from surgical biliary diversion [73,74]. Nasobiliary drainage may help to select potential responders to biliary diversion [75]. So far, clear genotype-phenotype correlation data are missing and remain to be defined in order to identify those PFIC1-2 patients who could benefit from UDCA or biliary diversion [3,60]. Preliminary data suggest that PFIC2 patients with p.D482G or p.E297G mutations may respond well to biliary diversion [60].

If these therapies fail, liver transplantation represents the only alternative [76]. In our experience, extrahepatic features, such as diarrhea, liver steatosis and short stature sometimes associated with PFIC1, do not improve or may be aggravated after successful biliary diversion or liver transplantation [9]. Chronic diarrhea may become intractable when biliary bile salt secretion is restored after liver transplantation [6,9,20]. It is often associated with severe liver steatosis and/or steatohepatitis that may lead to cirrhosis with time and to indication to retransplantation. Liver steatosis and diarrhea may recur after retransplantation (E. Jacquemin, personal data). Diarrhea might be favorably managed by bile adsorptive resin treatment [9,20].

In the future, therapies such as cell, gene or specific targeted pharmacological therapies (*i.e. *FXR inducers, chaperone drugs), might represent an alternative therapy for all types of PFIC [27,60,77-79]. In PFIC2, it is uncertain whether hepatocyte transplantation or gene therapy with modified hepatocytes represents a good therapeutic approach. Indeed, with this approach, it may be a risk to leave premalignant liver cells in place, especially in patients with severe biallelic *ABCB11 *mutations [80].

## Prognosis

In addition to the natural history and complications of PFIC1-3 (portal hypertension, liver failure, cirrhosis, hepatocellular carcinoma, extrahepatic manifestations), children with PFIC1-3 are theoretically at risk of developing further in the disease course, biliary stones, drug-induced cholestasis and ICP. Girls under UDCA therapy who reach adulthood with their native liver must not stop UDCA during pregnancy because of the risk of developing severe ICP as seen in a previously reported patient [53] who became pregnant (personal data Dr N. Ganne-Carrié, Hepatology Unit, Jean Verdier Hospital, Bondy, France).

Patients with BSEP deficiency, especially those with biallelic truncating mutations, are at considerable risk for hepatobiliary malignancy (15% may develop hepatocellular carcinoma or cholangiocarcinoma) [[25,80] and E. Jacquemin personal data]. These findings justify close monitoring of hepatocellular carcinoma (At least, serum alpha fetoprotein dosage every 6 months and liver ultrasonography every year) in PFIC2 patients from the first year of life.

## Unresolved questions

In very rare PFIC patients (< 10%), only one mutated allele or no mutation is identified. This can be explained by mutations that may map to regulatory sequences of the genes. A gene involved in the transcription of PFIC genes (*i.e. FXR*) or in protein trafficking could also be involved [81,82]. It is also possible that other unidentified genes involved in bile formation may be responsible for the PFIC1-3 phenotypes. Furthermore, it may be hypothesized that combined heterozygous mutations for two genes (*i.e. MDR3 *and *BSEP*) lead to PFIC like phenotype [49]. An interesting possibility is also that in a heterozygous state, the mutated protein may have a dominant negative effect on protein expression/function [83]. Modifier genes and environmental influences could play a role in the expression of PFIC [58].

After liver transplantation the possible "recurrence of PFIC" on liver grafts due to alloimmunization of the recipient against the BSEP, MDR3 or FIC1 proteins of liver donor remains a theoretical matter of debate. It is hypothesized that PFIC patients with a severe mutation leading to absence of the gene product would be immunologically naive for the FIC1, BSEP or MDR3 gene products. In this context, alloimmunization could occur after liver transplantation. To our knowledge, this complication has not been reported so far after liver transplantation [76,84] but we have observed a case of an PFIC2 male patient who experienced an unexplained severe bout of pure hepatocellular cholestasis resembling PFIC2 after cadaveric liver transplantation (E. Jacquemin, personal data). In addition, in case of parental living donor transplantation, it could be expected that the heterozygous status of the liver graft leads to a predisposition for developing lithiasis or cholestasis favored by immunosuppressive drugs [33] that may interfere with canalicular protein function, as we have seen in a PFIC2 patient (E. Jacquemin, personal data). Nevertheless, this complication is likely to be very rare, since it has not been reported in a broad series of living donor transplantation for PFIC [84].

## Experimental perspectives

As already mentioned above, studies performed in cell lines have determined the effects of some PFIC gene mutations (*ATP8B1 *[17-19], *ABCB11 *[26,28,29], *ABCB4 *[41,43,44]). This should allow testing of some drugs that may restore, at least partially, the function or the targeting of a mutated protein [27,78,79]. Besides these cell studies, mouse models of PFIC1-3 have been created by genetic engineering [85-87]. Mainly, the PFIC1-2 models have demonstrated that the phenotypes of the mice were much less severe than in humans. Although the phenotypes were aggravated by supplementation with cholic acid, the main bile salt in humans, this was not sufficient to induce liver injury comparable to that seen in PFIC1-2 patients. The likely reason for this is that mice, unlike humans, may easily detoxify hydrophobic bile salts by hydroxylation, *via *the cytochrome P450 enzyme system, resulting in a less cytotoxic bile salt pool. Therefore, it would be interesting to generate new mouse models that closely resemble PFIC1-2. Recently, cytochrome P450 knockout mice have been generated [88]. It is expected that crossing PFIC mouse models with mice with a disrupted liver cytochrome P450 oxidoreductase gene will generate mice sensitive to human bile salts. Such new mouse models could develop liver diseases that closely reproduce PFICs and will allow better experimental studies of the pathophysiology of PFICs and test the effects of treatments.

## List of abbreviations

PFIC: Progressive familial intrahepatic cholestasis; GGT: gamma-glutamyltransferase; BSEP: bile salt export pump protein; UDCA: Ursodeoxycholic acid; ICP: intrahepatic cholestasis of pregnancy; BRIC: benign recurrent intrahepatic cholestasis; ASBT: apical sodium bile salt transporter; CFTR: cystic fibrosis transmembrane conductance regulator; NBFs: nucleotide binding folds; MDR3: Class III Multidrug Resistance; P-gp: P-glycoprotein; MRS: Magnetic Resonance Spectroscopy; FXR: farnesoid X receptor.

## Competing interests

The authors declare that they have no competing interests.

## Authors' contributions

The authors equally contributed to this review article. They read and approved the final version of the manuscript.
